# Enhancing coral recruitment through assisted mass settlement of cultured coral larvae

**DOI:** 10.1371/journal.pone.0242847

**Published:** 2020-11-24

**Authors:** Dexter W. dela Cruz, Peter L. Harrison

**Affiliations:** 1 Marine Ecology Research Centre, School of Environment, Science and Engineering, Southern Cross University, Lismore, New South Wales, Australia; 2 The Marine Science Institute, College of Science, University of the Philippines, Diliman, Quezon City, Philippines; Academia Sinica, TAIWAN

## Abstract

The escalating rate at which coral communities are declining globally requires urgent intervention and new approaches to reef management to reduce and halt further coral loss. For reef systems with limited natural larval supply, the introduction of large numbers of competent coral larvae directly to natural reef substrata provides a potentially useful approach to replenish adult coral populations. While few experiments have tested this approach, only one experiment has demonstrated its long-term success to date. Given the differences in life-history traits among corals, and different sensitivities of larvae to abiotic and biotic factors, coupled with the dynamic nature of post-settlement survivorship and recruitment processes, trials of the larval enhancement technique with larvae of different coral species are needed to test the broader applicability and viability of this approach. Accordingly, in this paper we examine the applicability of the larval enhancement technique to restore a population of *Acropora loripes* in the Bolinao-Anda Reef Complex, Pangasinan, northwestern Philippines. Larvae were cultured ex situ following spawning of collected *A*. *loripes* colonies in June 2014. Competent larvae were transported to degraded reef areas and approximately 300,000 larvae were introduced in each of three 6 × 4 m plots directly on the reef. Fine mesh enclosures retained the larvae inside each treatment plot for five days. Three adjacent 6 × 4 m plots that served as controls were also covered with mesh enclosures, but no larvae were introduced. Each plot contained ten 10 × 10 cm conditioned settlement tiles cut from dead tabulate *Acropora* that were used to quantify initial larval settlement. After allowing larval settlement for five days, mean settlement on tiles from the larval enhancement plots that were monitored under stereomicroscopes was significantly higher (27.8 ± 6.7 spat per tile) than in control plots, in which not a single recruit was recorded. Post-settlement survivorship and growth of spat and coral recruits on tiles and reef substrata inside the experimental plots were monitored periodically for 35 months. After 35 months, the mean size of each of the remaining 47 *A*. *loripes* coral colonies surviving on the reef substrata was 438.1 ± 5.4 cm^3^, with a mean diameter of 7.9 ± 0.6 cm. The average production cost for each of the surviving *A*. *loripes* colonies at 35 months was USD 35.20. These colonies are expected to spawn and contribute to the natural larval pool when they become reproductively mature, thereby enhancing natural coral recovery in the area. This study demonstrates that mass coral larval enhancement can be successfully used for restoring populations of coral species with different life-history traits, and the techniques can rapidly increase larval recruitment rates on degraded reef areas, hence catalysing the regeneration of declining coral populations.

## Introduction

The increasing rate at which coral communities are declining globally requires urgent intervention and new approaches to reef management to reduce and halt the loss, and increase coral cover and diversity on degraded reefs [1, 2]. Therefore, active management interventions and novel restoration approaches are needed to help in reef recovery at local, regional and global scales; recovery that is currently unlikely to occur based on natural recruitment processes alone. For these reasons, management and restoration tools are increasingly considered as essential to mitigate coral reef degradation caused by anthropogenic disturbances [3–7].

In the initial stages of coral restoration research, direct coral transplantation gained popularity as one approach to rapidly increase coral cover by re-attaching coral fragments or whole colonies to degraded reefs [5, 8, 9]. As an alternative, an intermediate step of rearing coral nubbins in different types of nurseries increased the number of transplant materials from a few source colonies [10–12]. While this grow-out nursery phase increases the size and robustness of coral transplants, which may lead to higher post-transplantation survival rates [13–15], it also increases production costs from hatchery and nursery facility construction, grow-out costs, and outplanting costs [5, 16].

More recently, sexual larval propagation has become more widely used in restoration studies. This approach aims to increase recruitment rates, coral cover [17], and genetic diversity that may improve coral adaptive and evolutionary potential, and increase resilience in depleted coral populations [18–20]. Using this approach, millions of sexually derived coral larvae can be sourced from sexually mature coral colonies from ex situ spawning in a controlled hatchery facility [21, 22], or in situ by using spawn collectors placed on top of individual corals [23], or from natural coral spawn slicks [24–26]. Typically, competent larvae are then settled on artificial substrata and kept in land- or ocean-based nurseries before they are outplanted on the reef [5]. For example, Villanueva et al. [21] cultured *Acropora valida* larvae that were settled on coral rubble. After a few months of hatchery rearing, the juvenile corals on substrata were directly attached to reef sites using adhesive. The same approach was employed by Baria et al. [27] where larvae of *A*. *granulosa* were settled on artificial substrata in the hatchery and subsequently transplanted to the reef. The cost of each juvenile in the nursery phase was 2.79 USD but increased to 20.01 USD each after transplantation because of additional outplanting costs and subsequent coral mortality [27].

To reduce these labor-intensive and costly grow-out and outplanting phases, coral larvae can be immediately outplanted shortly after the completion of metamorphosis and onset of skeleton formation [28]. For example, *Acropora palmata* settlers outplanted to the reef at the age of two weeks were 7 times more likely to survive and were 25 fold less costly to produce than conspecifics kept within a land-based nursery for 2.5 years [28, 29]. In addition, new tetrapod-shaped substrates have been developed that can be “seeded’ onto the reef in much less time and 5–18 fold lower costs compared with traditional outplanting techniques because their geometry allows them to be wedged within reef crevices, thus avoiding the need for adhesives and nails for attachment [28, 29]. Nonetheless, whether corals are derived from asexual or sexual propagation, the transplantation of corals remains costly, labor-intensive, time consuming and in many cases, has failed to restore self-sustaining coral populations and associated ecological functions at the restoration site [30, 31].

A less commonly used approach that avoids the need for manual transplantation of corals settled onto artificial substrates is the introduction of large numbers of competent larvae directly onto reef areas. This approach is however still underdeveloped and poorly explored, as most previous studies have either provided accounts of larval release in the sea without quantifying subsequent settlement and recruitment rates [32], or experiments were carried out in small areas (1 × 1–2 m) using artificial substrata, and lacked long-term monitoring or direct monitoring on the natural reef substrata [24, 33]. Overall, these early experiments did not demonstrate an increase in adult coral cover as a result of increasing larval supply.

Recent work by dela Cruz and Harrison [17] provided the only case study demonstrating a significant effect of enhancing larval supply on subsequent recruitment and increased adult coral cover on degraded reef areas, and re-established a breeding population within three years. About 400,000 *A*. *tenuis* larvae were released into four 4 × 6 m plots on degraded reef areas in the northwestern Philippines that were temporarily enclosed with fine mesh matting during the larval settlement period. Initial larval settlement was high and juvenile survivorship began stabilising after five months. At least two colonies per m^2^ survived on the available natural reef substrata and these grew rapidly and spawned successfully at the age of three years, thereby completing the coral life cycle.

Acknowledging the gap between the small scale of most current restoration attempts and current state of degraded coral reefs worldwide, there is increasing emphasis on the need to upscale restoration interventions that can create persistent, viable and ecologically functioning reef communities [5, 7, 34]. Many millions of larvae are produced after major coral spawning events [35, 36] that can be used for restoration, and there are well-established techniques for ex situ larval culture of different coral species [18, 34]. Therefore, there are good opportunities for improving and modifying the larval enhancement techniques for coral restoration interventions to cover larger areas of damaged but recoverable reefs, where larval supply is now limited.

Different coral species may respond differently to specific coral restoration methods. For example, Miller [37] had found that the survivorship of transplanted *Acropora palmata* recruits cultured from larvae was generally higher than for *Orbicella faveolata* recruits in the Caribbean. Given the differences in life-history traits among corals, and different sensitivities of larvae to abiotic and biotic factors (e.g., temperature, water quality, conditions on settlement substrata etc.), coupled with the dynamic nature of post-settlement survivorship and recruitment processes [34, 38–40], repeated trials of the larval enhancement technique with larvae of different coral species are needed to verify this approach.

Accordingly, in this paper we examine the broader applicability of the larval enhancement technique using larvae of *Acropora loripes*. This is a relatively common and widely distributed coral species in the Indo-Pacific region [38, 41]. As of 2014, it was listed as a “near threatened species” by the IUCN due to extensive population reduction [42]. *Acropora loripes* has a different life-history compared to *A*. *tenuis*, and grows more slowly and is likely to reach reproductive maturity later [38]. These corals are also phylogenetically divergent and belong in different clades [43], and *A*. *tenuis* is an “early spawner” that spawns just after sunset, and *A*. *loripes* is a “late spawner” that spawns a few hours after sunset [44]. Previous laboratory experiments have shown that both species exhibit high fertilization rates (>90%) and larval settlement rates (>50%) [45, 46]. However, *A*. *lorip*es showed better survivorship than *A*. *tenuis* in laboratory experiments [45, 46]. Although it is one of the most common corals used for the aquarium trade [47], it is not a popular experimental species and very few researchers have used it for coral restoration intervention or ex situ coral culture [45, 48].

## Materials and methods

### Experimental design

This experiment was designed to test the effect of supplying large numbers of *Acropora loripes* coral larvae on replicate degraded reef areas and to quantify and compare initial larval settlement, and subsequent post-settlement survivorship and growth patterns of recruits for 35 months. The following methods were adapted from dela Cruz and Harrison [17].

### Site selection

**Fig 1:**
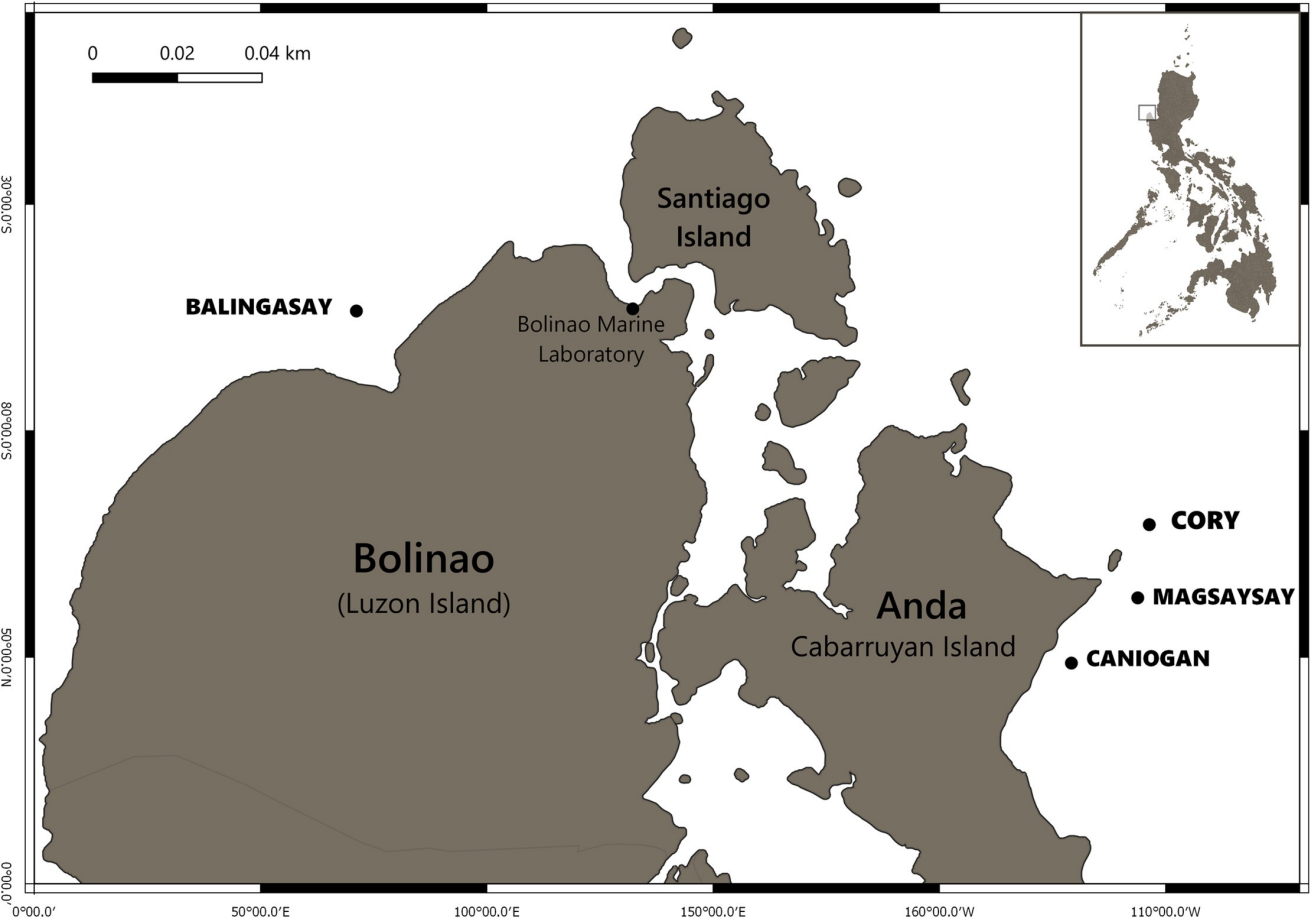
Locations of the experimental larval-enhanced and control plots (Magsaysay reef) and the source of *A*. *loripes* colonies (Caniogan and Balingasay reefs) in the Bolinao-Anda Reef Complex, northwestern Philippines.

The *A*. *loripes* larval enhancement experiment was conducted on degraded reef areas at Magsaysay reef, Anda, Pangasinan (16°19’36” N, 120°02’01” E) at 4–5 m depth (Fig 1). The *A*. *loripes* plots were located between 5 to 15 m away from the plots used for the previous *A*. *tenuis* larval enhancement experiment a year earlier [17]. The same reef restoration area was selected to enable more direct comparison of the results from these two separate experiments using two different coral species. This 14.8 ha shallow reef area is an important fishing ground for people in the coastal community of Anda and nearby municipalities [49–52]. The major causes of destruction of the reef areas were blast fishing that no longer occurs, occasional typhoons and a large Crown-of-Thorns starfish infestation that was observed in 2007 [49].

A total of six 6 × 4 m plots were haphazardly selected and demarcated using steel bars, and three plots were provided with larvae (larval enhancement) and the other three plots served as controls without cultured larvae. The plots were carefully searched for any *A*. *loripes* adult colonies and recruits (<5 cm) prior to the larval enhancement activity, and only one adult colony of *A*. *loripes* was present within one of the three control plots. Prior to the larval enhancement experiment, photographs of the plots were taken using a 1 × 1 m frame quadrat and photos were analyzed using CPCe [53] to quantify benthic cover and to determine the status of the benthic community. A total of 10 random points were generated and scored in each of 24 frames taken in each of the plots [54].

**Fig 2:**
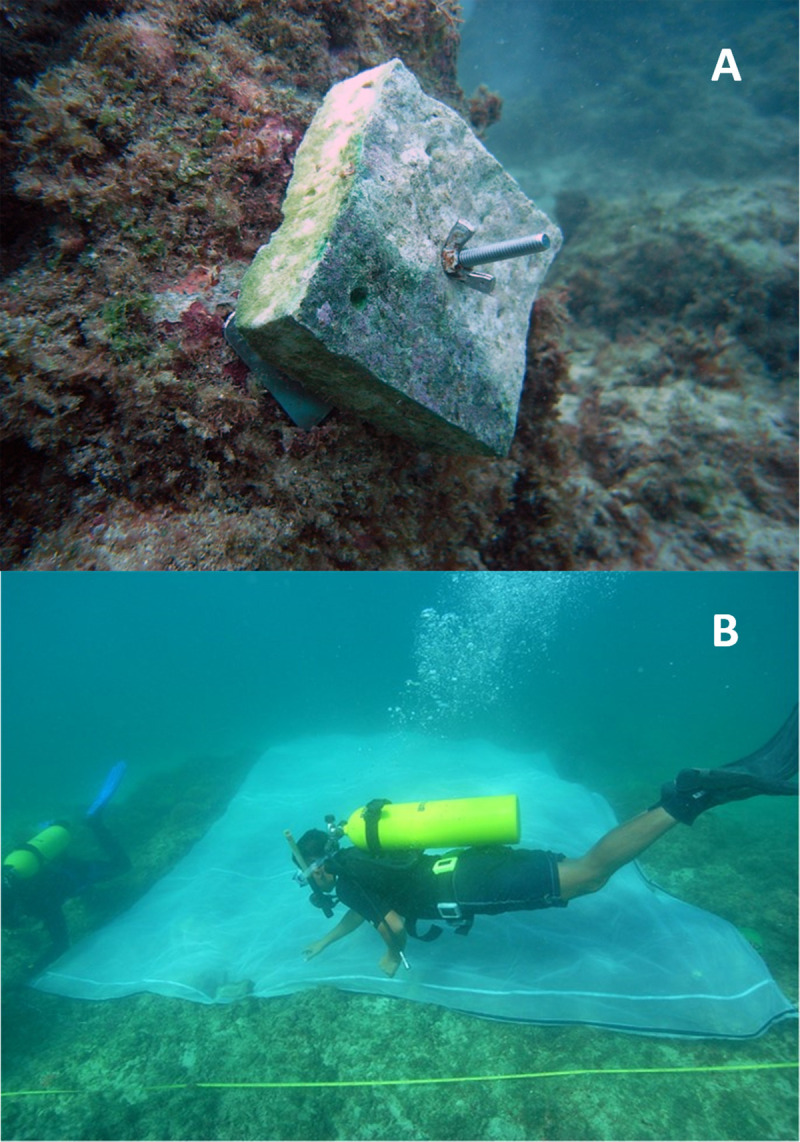
(a) Sample dead coral tile deployed in larval-enhanced and control plots used to determine initial larval settlement. (b) The mesh matting placed onto the larval enhancement plot.

Ten 10 × 10 cm ‘natural’ settlement tiles (cut from dead table *Acropora*) with varying thickness (mean 3–4 cm) were deployed inside each plot just prior to the larval enhancement experiment, and each tile was identified with a coded tag (Fig 2A). The coral settlement tiles were designed to be easily removed and re-attached to the reef to allow more accurate, repeated monitoring of initial settlement and subsequent survival rates, especially during earlier stages of this study when recruits were very small and not visible with the naked eye. The total surface area of each tile was estimated to be about 360 ± 3.7 cm^2^ based on the 3D scanned tiles used in dela Cruz and Harrison [17]. The tiles were directly attached to the substrata using stainless base plates [55]. Dead tabular *Acropora* plates with naturally growing crustose coralline algae (CCA) used in the production of the tiles were collected at the intertidal zone beside Cory reef rubble bar (Fig 1). Recruitment tiles were biologically conditioned for a month in hatchery tanks at the Bolinao Marine Laboratory (BML) of The Marine Science Institute (University of the Philippines) with flow-through seawater and aeration to promote further CCA growth prior to deployment. This ex situ conditioning of tiles avoided the natural settlement of coral larvae onto the tiles prior to the experiment. This was confirmed by examining the tiles under stereomicroscopes before the experiment to ensure that no coral recruits were present.

### Larval culture

*Acropora loripes* was used for this experiment. Colonies are usually semi-circular horizontal plates, and do not grow more than a metre across. *Acropora loripes* colonies have varying growth forms ranging from upright bushes to thick plates. This species is found in most reef environments from shallow reef habitats to 25 m deep, especially on reef slopes protected from wave action. In the Bolinao-Anda Reef Complex (BARC), northwestern Philippines, *A*. *loripes* can be found from shallow (2 m) to deeper reef areas (9 m).

A week before the full moon in June 2014, twenty-five gravid colonies of *A*. *loripes* (diameter of 7–15 cm) were collected from ~2–7 m deep reef areas on Caniogan reef and Balingasay reef, Bolinao and Caniogan, Anda, Pangasinan (Fig 1). The collections of corals were allowed under the Prior Informed Consent Certificate issued by the municipalities of Bolinao (document number 320:2013) and Anda (document number 165:2012). Colonies were confirmed to be gravid by carefully breaking a few branches to check for the presence of pigmented (pink to red) maturing oocytes [35]. Collected gravid colonies were carefully transported in polyethylene bins with seawater to the BML hatchery facility for ex situ observation of spawning and gamete collection.

Coral ex situ spawning, gamete collection and larval culture followed standard protocols [21, 56–59]. Colonies were held in concrete tanks with flow-through seawater and aeration. Spawning was checked nightly by periodic monitoring of colonies from 1930 to 2130 h. Seawater flow and aeration were turned off during each monitoring period to prevent disturbance to corals that may delay spawning. Spawning occurred 120 to 150 minutes after sunset on June 16 and 17, 2014 (3 and 4 nights after the full moon) and gametes from these spawning events were collected and used for the experiment.

Spawned egg-sperm bundles were skimmed off the water surface using 400 mL plastic cylindrical containers. The collecting container was slowly submerged into the water surface to allow the egg-sperm bundles and small amounts of seawater to flow in. Collected egg-sperm bundles were then transferred to a fertilization polyethylene tank containing 10 L of 1 μm filtered seawater. Gamete bundles were gently agitated to facilitate gamete separation and subsequent cross-fertilization. After 1 h, excess sperm were removed (sperm-washing) by slowly opening the valve located near the bottom of the fertilization tanks. The valve was closed before the water level with floating eggs reached the outflow, and new filtered seawater was slowly added. Washing was done thrice to remove excess sperm that may degrade water quality during larval culture [60]. Subsamples of embryos and eggs were collected using 15 mL tubes and examined under a stereomicroscope after a further hour to determine the percentage fertilization.

Developing embryos were transferred into 11 rearing tanks each containing 1,000 L of seawater. Fresh filtered seawater was added every day (~50 L) to maintain the developing larvae in a healthy condition. Aeration was supplied 24 h after fertilization. At 4 d post-fertilization, an estimated 939,000 ± 29,000 SEM competent larvae were collected using plankton net sieves (60 μm mesh pore) and transferred to a temporary holding tank. The total number of larvae was estimated by thoroughly mixing and dispersing larvae throughout the water column then taking three replicate 60 mL samples. The larvae were then distributed equally into 40 × 50 cm strong plastic bags. Oxygen was supplied to each bag before it was sealed for transport to the field for the larval enhancement experiment.

### Coral larval enhancement

The in situ larval settlement enclosure system used by dela Cruz and Harrison [17] was used for this experiment (Fig 2B). Just prior to the experiment, corals inside the plots were temporarily covered with Amazon™ plastic mesh with pore size of 1 cm to avoid the matting from ripping or damaging coral tissues, and the mesh was removed after the 5-day settlement period. The larval mesh enclosures measured 6 × 4 m and were composed of a layer of organza cloth (100–150 μm mesh pore sizes) sewn onto a second layer of nylon net (1 mm openings). This matting assembly can effectively retain *Acropora* larvae whose diameters are 300–500 μm [56, 61]. To firmly hold the matting on the reef substrata and prevent the larvae from drifting out of the plot during the settlement period, cylindrical lead weights (1.75 × 4.00 cm; 20 g) were inserted along the matting edge. Additional steel bars, driven into the reef substrata, were added on each corner of the matting.

Approximately 300,000 *A*. *loripes* larvae were added to each of three replicate 4 × 6 m mesh enclosures on reef plots, with three replicate control plots that were not provisioned with cultured larvae. Larval mesh enclosures were removed from each of the six plots after the 5-day settlement period and the settlement tiles were carefully collected and transported to the BML facility while submerged in seawater, where the initial number of settled larvae on each tile were recorded under stereomicroscopes. Tiles were then returned to their correct location and orientation within each reef plot, and the survival and growth of settled spat on tiles was monitored at 2, 4, 6, 8, 10, 18, 21, 23, 25, 27, 31, 33 and 35 months after settlement.

### Coral recruits on natural reef substrata

Surviving *A*. *loripes* recruits on natural reef substrata were visible for in situ monitoring at 8 months after settlement. At this time point, each juvenile colony was identified with a numbered aluminum tag placed nearby to facilitate repeated in situ monitoring of growth and survival at 8, 10, 18, 21, 23, 25, 27, 31, 33 and 35 months after settlement.

In situ growth monitoring commenced at this time, with the length (*l*), width (*w*) and height (*h*) of each of the juvenile corals on recruitment tiles and natural substrata measured using calipers. Mean planar diameter was calculated from the maximum and minimum diameters measured for each colony. The ecological volume (EV) was calculated using the volume formula: EV = πr^2^*h*, where r = (*l*+*w*)/4 [31]. Growth rates (ecological volume change per month) were also calculated, using the formula G_r_ = [EV_f_−EV_i_]/m, where G_r_ is standardized growth rate, EV_f_ and EV_i_ are final and initial mean ecological volumes, respectively, and m is the number of months elapsed [62].

The onset of sexual reproduction in *A*. *loripes* colonies that recruited onto coral tiles and natural reef substratum was monitored at age 23 and 35 months by carefully breaking small branches to check for the presence of gametes [35]. The broken branches were then gently wedged back between the fragments of the colony to avoid loss of branches and tissues. These gamete monitoring periods were completed just prior to the predicted potential spawning periods after 2 and 3 years of growth [38].

### Coral production cost analysis

To estimate the cost of producing sexual coral recruits from this study, the costs were categorised and then summed for all materials and infrastructure, boat hire and fuel, diving and labour for gravid coral collection (using different wage rates for different skill levels as prescribed by the Department of Science and Technology Grant-in-Aid personnel), spawning and larval rearing, site preparation and capital costs for the larval mesh enclosures. To estimate the cost per coral colony produced at different ages, the total cost was divided by the total numbers of juvenile corals alive at 8 months and at three years of age in the three larval enhancement plots. Costs were in Philippine Pesos and were converted to US Dollars.

### Statistical analyses

Data are reported as mean values ± standard error of the means. The three larval enhancement sites and the three control sites were used as statistical replicates (N = 3), with data from the ten tiles in each site averaged to quantify mean initial settlement rates, and subsequent growth and number of surviving recruits at age 35 months.

The variability in the benthic cover composition (e.g., sand, rubble, macroalgae, coral) between larval enhancement and control plots prior the larval enhancement experiment was analysed using Analysis of similarities (ANOSIM). Significant differences in the initial settlement patterns on tiles between larval-enhanced and control plots after five days of settlement was tested using One-way ANOVA. A post-hoc Tukey’s HSD test was conducted to determine any significant differences in settlement patterns among tile surface types (i.e., top, bottom, sides).

Survivorship of coral recruits on different surfaces of tiles was analysed using survival analysis, a non-parametric pairwise comparison test based on the Kaplan–Meier function [63]. The same analysis was used to determine any significant difference in survival patterns of juvenile corals on natural substrata and on tiles from nine to 35 months after the larval enhancement. Significant increases in growth (ecological volume and mean diameter) of juvenile corals through time were determined using Repeated Measures ANOVA. One-way ANOVA was used to compare growth rates (expressed as ecological volume and mean diameter) of juvenile corals on recruitment tiles versus growth rates on natural substrata. To determine if the assumptions of ANOVA were met, Shapiro-Wilk normality tests and Levene’s test of homoscedasticity were used on each independent variable. Sphericity tests were also conducted prior to running all repeated measure ANOVAs.

## Results

### Benthic cover and reef condition

**Fig 3:**
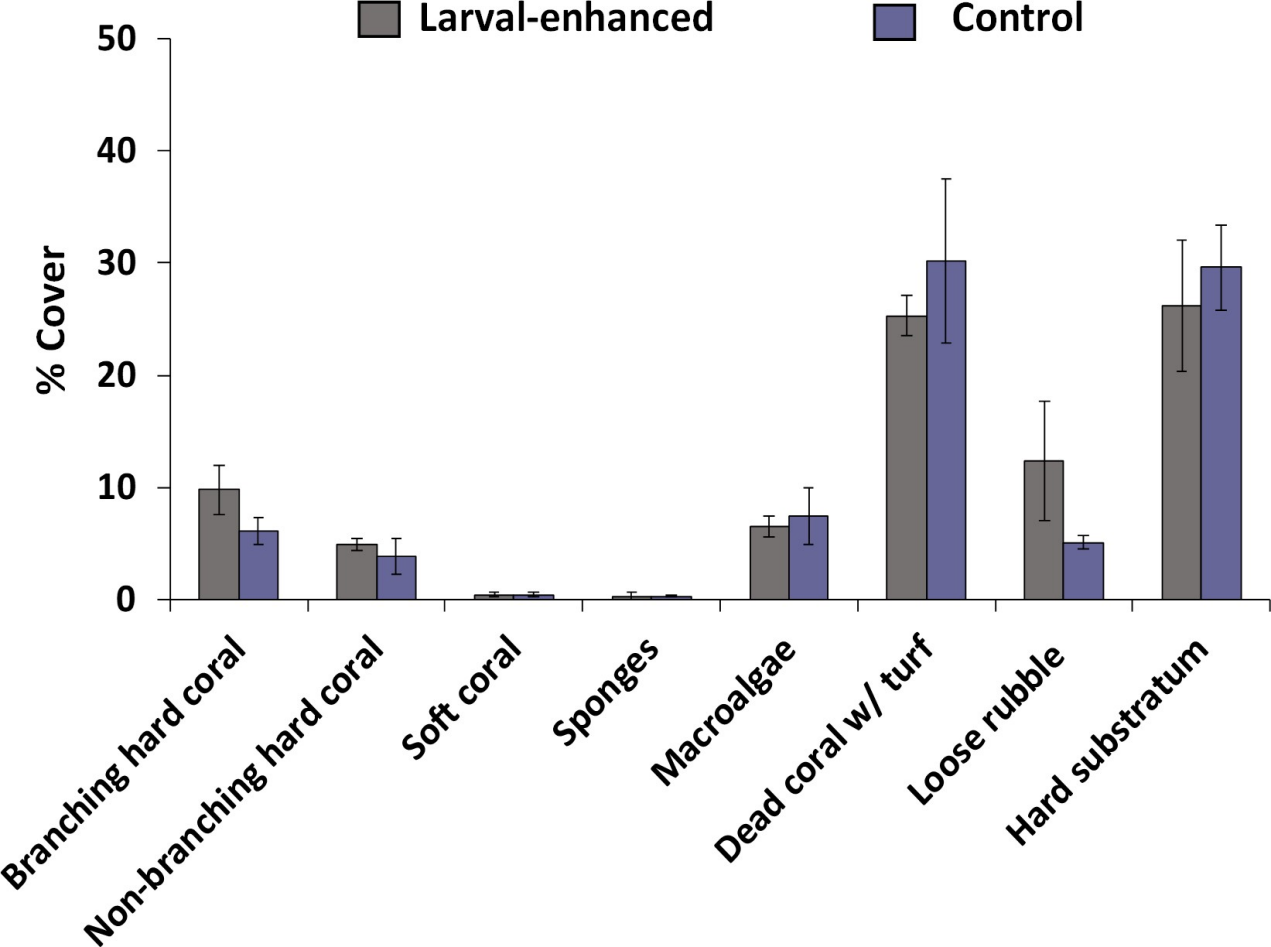
Mean percentage cover of benthic categories in larval-enhanced (N = 3) and control (N = 3) plots before the experiment. Total available area on each plot for coral settlement, which is about 8 m^2^, is a combination of dead hard coral and rubble. Error bars are ± SEM.

Larval-enhanced and control plots had comparable benthic cover compositions before the larval enhancement experiment (ANOSIM, *R*: -0.11, p = 0.8). The benthic communities within the plots corresponded to the category “very poor coral cover” (*sensu* Wilkinson) [64] comprising of 12.3 ± 1.9% mean live coral cover (branching + non-branching hard corals; Fig 3). Other biotic benthic categories included low cover of soft coral, sponges, macroalgae, with dead coral covered with turf algae comprising 27.8 ± 4.5% mean cover. Plots also contained 36.7 ± 3.8% mean cover of abiotic dead coral rubble and dead hard coral substrata (Fig 3), with about 8 m^2^ (out of the 24 m^2^) of available substrata (dead hard coral + rubble) per plot for the larvae to settle.

### Larval development and initial settlement

**Fig 4:**
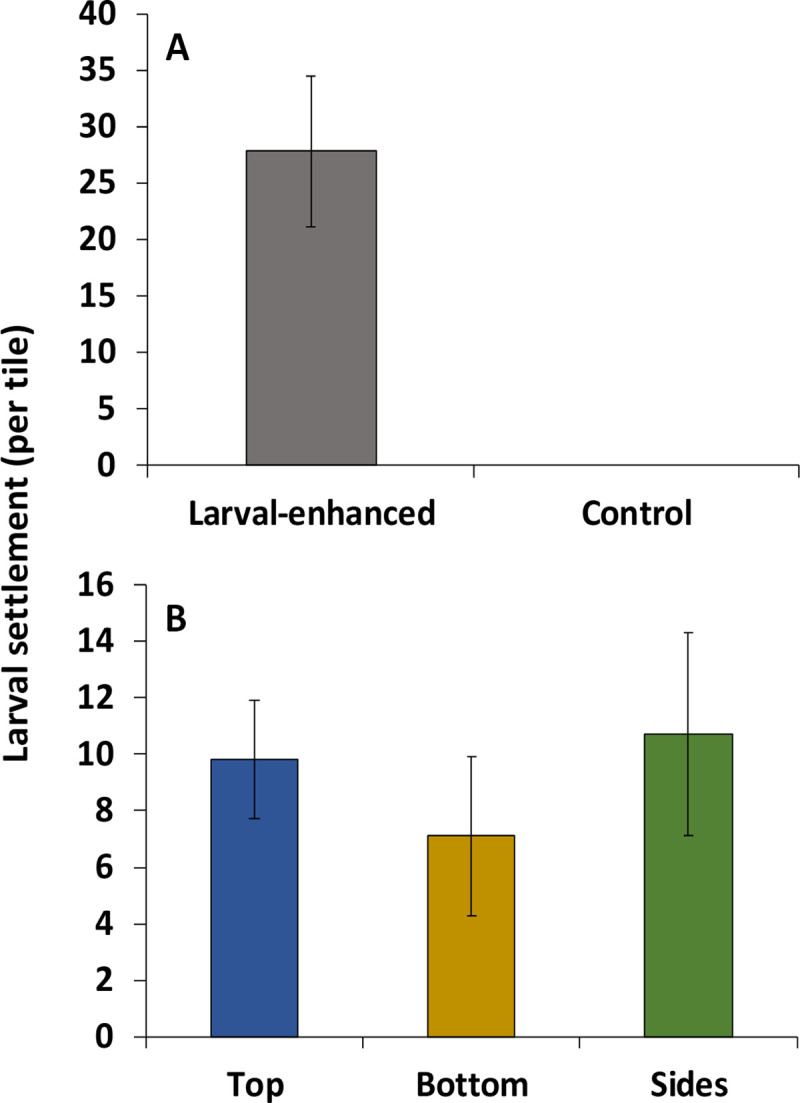
(a) Mean initial *A*. *loripes* larval settlement on all tile surfaces in the larval-enhanced (N = 3) and control (N = 3) plots after 5 days, and (b) mean settlement on the different surfaces of tiles in the larval-enhanced plots (N = 3). Means are for ten tiles per plot and are averaged among the three replicate plots. Error bars are ± SEM.

A total of 834 *A*. *loripes* spat settled on the thirty 10 × 10 cm biologically conditioned natural *Acropora* skeleton tiles that were attached to reef surfaces in the larval enhancement treatment plots during the 5-day settlement period. Mean total settlement on the sets of ten tiles in each of the three larval enhancement treatment plots was 278 ± 67.3 spat, which was significantly higher than for control sites in which no *A*. *loripes* spat settled (Fig 4A).

Mean larval settlement on top surfaces of the tiles was 98 ± 21.4, with 71.3 ± 28.3 and 108.7 ± 36.2 mean settlement on the bottom and side surfaces, respectively (Fig 4B). No significant differences were found between the mean number of settled spat among the surfaces of the settlement tiles (*F*_*7*,*1*_ = 1.67, p = 0.55).

### Survivorship

**Fig 5:**
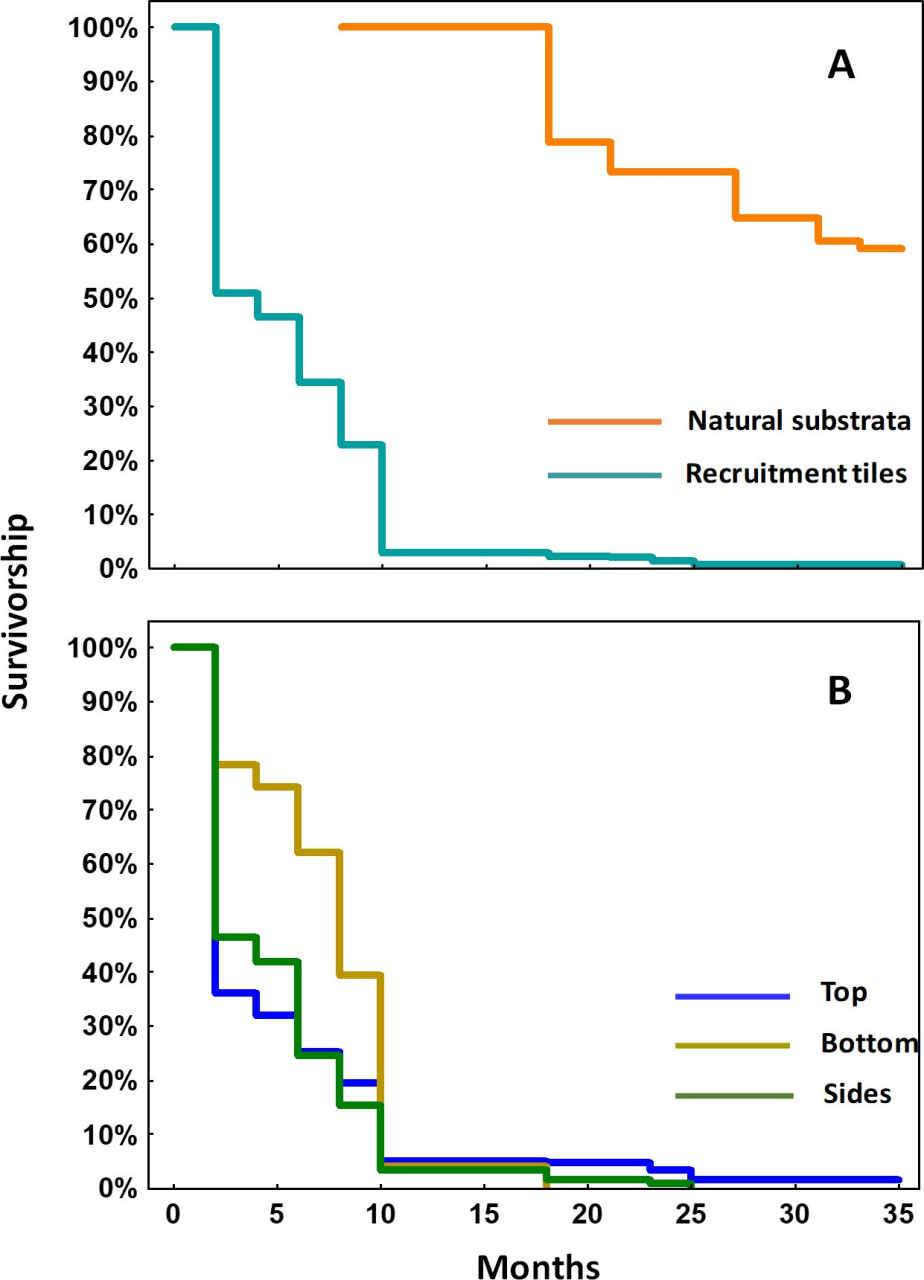
Kaplan-Meier survivorship for 35 months for (a) *A*. *loripes* recruits settled on tiles (N = 834), and for visible recruits on natural reef substrata starting at 8 months post-settlement (N = 72) (b) *A*. *loripes* recruit survivorship on different tile surfaces (N = 834).

Monitoring of coral spat on tiles showed the expected decline in survivorship after the larval settlement period. An almost 50% decline in survivorship on tiles was recorded after 2 months (Fig 5A). Survivorship continued to decline until 10 months, then few mortalities were recorded on tiles during subsequent monitoring periods up to 35 months (Fig 5A).

After eight months, the previously cryptic juveniles that had settled on the natural reef substrata became visible recruits at 1.2 ± 0.04 cm mean diameter (1.1 ± 0.2 cm^3^ mean ecological volume) for in situ growth and survivorship monitoring (Fig 5A). Eight months after settlement, a total of 72 *A*. *loripes* recruits were recorded on the natural reef substrata in the three larval enhancement plots, and a total of 23 recruits survived on the settlement tiles (95 juveniles in total).

For corals that settled on the natural reef substrata, 100% survivorship was recorded during the first 8 months after they became visible, and survivorship declined slowly and reached 59% survivorship on the last monitoring period in May 2017, 35 months after settlement (Fig 5A). Survivorship of recruits on tiles from June 2014 to May 2017 varied significantly among tile surfaces (χ^2^ = 93.3, p = <0.01, Log-rank test; top = sides > bottom; Fig 5B). During the first 9 months, survivorship of juvenile corals on the bottom of the tiles was higher compared to top and side surfaces. However, from 10 months onward most of the juveniles surviving were from the top and sides of tiles, and all the remaining juveniles at the last monitoring period were from the top surface. The mean total number of surviving recruits on the ten tiles in each settlement site after 35 months was 1.7 ± 0.3, which equates to 4.6 ± 0.9 colonies per m^2^ of tile surface. The total number of surviving *A*. *loripes* on the natural reef substrata after 35 months was 42 colonies, with five surviving colonies on the tiles (47 surviving colonies in total).

### Recruit growth and onset of sexual reproduction

**Fig 6:**
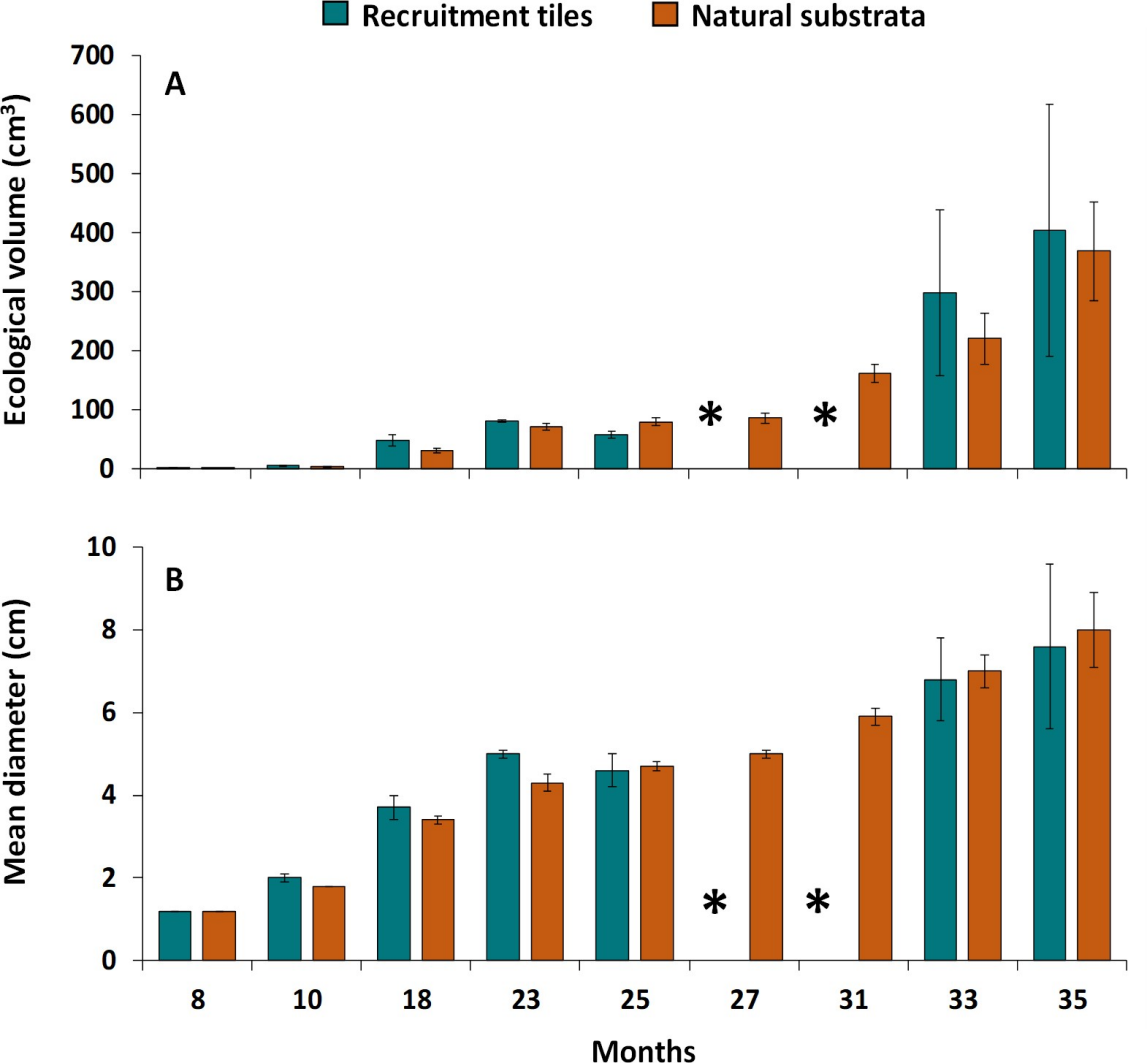
Mean volume (a) and mean diameter (b) of juvenile *A*. *loripes* on recruitment tiles and natural substrata inside the three larval-enhanced plots. * No data. Error bars are ± SEM.

**Fig 7:**
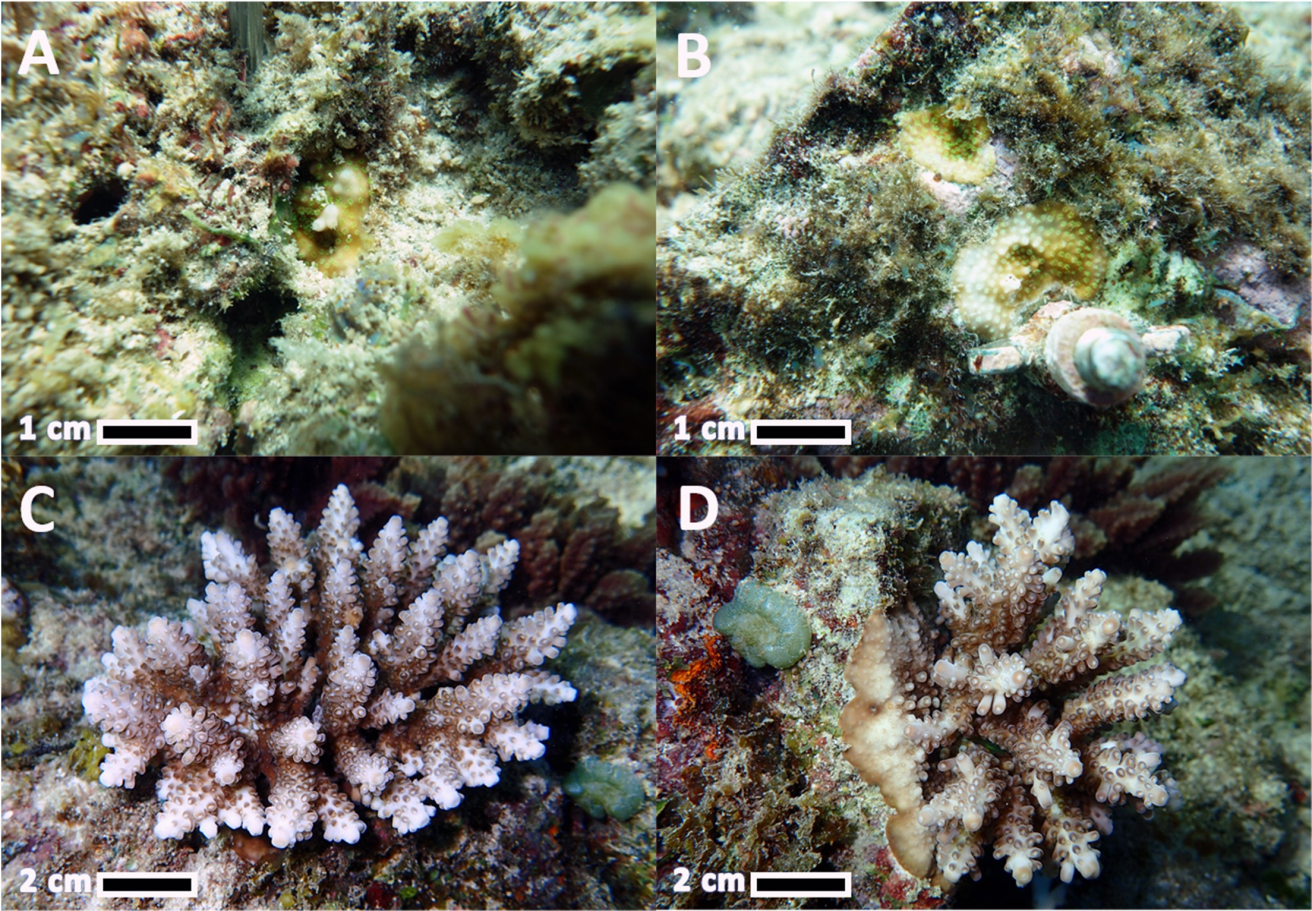
Representative *A*. *loripes* recruits at 8 months post-larval enhancement on (a) natural reef substrata and (b) on settlement tiles. Three-year-old *A*. *loripes* corals on (c) natural reef substrata and (d) on settlement tiles.

Initial mean volumes of juvenile corals on the natural substrata and on tiles 8 months after the larval enhancement activity were 1.1 ± 0.1 cm^3^ and 1.3 ± 0.9 cm^3^ respectively (Figs 6A, 6B, 7A and 7B). After 35 months, the mean volumes of corals on the tiles reached 438.1 ± 5.4 cm^3^ and 369.1 ± 8.4 cm^3^ for the corals on natural substrata (Figs 6A, 7C and 7D). The average volumes of corals on tiles and natural substrata did not differ significantly during any of the monitoring periods (Repeated Measures ANOVA; p = >0.05). Average growth rates of *A*. *loripes* recruits monitored on natural reef substrata from 8 months to 35 months reached 14 ± 3 cm^3^ mo^-1^ and were similar (*F*_*1*,*4*_ = 2.36, p = 0.71, ANOVA) to growth rates of recruits that settled on tiles (16 ± 5.7 cm^3^ mo^-1^). Thirty-five months after settlement, mean diameters of the colonies ranged from 2.2 cm to 13.9 cm with an average of 7.9 ± 0.6 cm (Fig 6B). Sampling of coral fragments for potential spawning periods after two and three years indicated that none of the colonies had become sexually reproductive.

### Production costs

The total production cost for the sexually derived *A*. *loripes* colonies in this study was USD 1654.00. The cost of producing the mesh mattings was divided into two because these mesh enclosures were re-used from the previous larval enhancement activity with *A*. *tenuis* [17]. This equates to a production cost of USD 17.41 for each of the 95 recruits alive at 8 months, and USD 35.20 for each of the 47 colonies surviving after 35 months (Table 1).

**Table 1:** Summary of costs for different activities related to larval enhancement and production of coral colonies.

Activities	Days	Total (USD)
I. Collection of gravid *A*. *loripes* colonies	1	
A. Materials (including SCUBA equipment rental)		74.32
B. Boat rental		105.40
C. Hired labor (2 personnel)		39.11
Sub-total		218.83
II. Hatchery work	10	
A. Facility and support system		9.10
B. Culture tanks and accessories*		10.40
C. Hired labor (2 personnel)		357.80
Sub-total		377.30
III. Larval enclosures production**	~15	
A. Materials		145.40
B. Hired labor (3 personnel)		103.56
Sub-total		248.96
IV. Site selection and preparation	1	
A. Materials (including SCUBA equipment rental)		171.82
B. Boat rental		105.40
C. Hired labor (3 personnel)		54.67
Sub-total		331.89
V. Larval enhancement activity		
A. Materials (including SCUBA equipment rental)	1	253.94
B. Boat rental		105.40
C. Hired labor (10 personnel)		118.00
Sub-total		477.34
**TOTAL**		**1654.32**
**Cost per colony 8 months post-larval enhancement**		**17.41**
**Cost per colony 35 months post-larval enhancement**		**35.20**

SCUBA gear hire, air tanks and boat rental from BML to the study site were based on 2013 and 2014 rates. The BML outdoor hatchery facility and support system costs include equipment (seawater pump, air blower, pipe assemblies, sedimentation tank), maintenance and electricity. Costs were originally estimated in Philippine Pesos (PhP) and converted to USD using the conversion rate: PhP 45 = USD 1.

*total value divided over 10 years of use

**total value divided by two uses.

## Discussion

Natural reef recovery following a disturbance depends on ample coral larval supply, successful settlement of larvae and survival to adult reproductive age [39, 65, 66]. Each of these coral life-history stages is a potential bottleneck that significantly affects coral population recovery. In previous decades, coral restoration interventions have primarily utilised juvenile and adult coral stages as transplant materials to immediately increase coral cover on degraded reefs [9]. Recent research has focused on sexual production of corals to increase genetic diversity and potential resilience [19, 26, 34], and the previous pilot study using *A*. *tenuis* larvae clearly demonstrated that supplying competent swimming coral larvae to settle directly on degraded reefs significantly increased coral recruitment [17]. The *A*. *tenuis* long-term reef experiment was the first to demonstrate that larval enhancement on degraded reef areas significantly increases settlement leading to increased recruitment and coral cover, and also re-established reproductively mature adult coral colonies within three years.

Over the past few decades, advances in ex situ larval culture research have improved the rearing and production of larvae for small scale laboratory assays and growing juvenile corals for transplantation [18, 22, 67, 68]. Larger scale ex situ larval culture of various coral species has also been used to rear millions of larvae for coral restoration experiments [17, 28, 33]. Ex situ larval culture may increase survivorship of settled larvae by carefully selecting stress tolerant, including heat-tolerant, breeding corals. Also, the ability to manipulate culture conditions using stress-hardening may produce larvae better adapted to warming environments, promoting better survivorship [6]. Furthermore, rearing large numbers of coral larvae from multiple coral colonies, whether in ex situ cultures or from natural coral spawn slicks, creates large pools of new genetic variants of corals. An advantage of the larval restoration approach is that these genetically diverse larvae are exposed to natural reef conditions during settlement, avoiding artificial selection pressures during settlement and early life stages compared with laboratory cultures that might prove to be maladapted to environmental conditions at the restoration sites. In the present study, 900,000 *A*. *loripes* larvae were successfully cultured ex situ in culture tanks and the larvae were readily transported and released into the reef plots where some *A*. *loripes* corals were observed to be growing nearby.

Unlike other *Acropora* species in Magsaysay reef (i.e. *A*. *cytherea*, *A*. *hyacinthus*, *A*. *muricata*, *A*. *sarmentosa* etc.) that recovered from stressors that these local reefs experienced during recent decades, only four colonies of *A*. *loripes* were recorded surviving in surveys on ~200 m^2^ of nearby reef area. With very few adult colonies to supply gametes or larvae to aid natural recruitment and reef recovery, *A*. *loripes* population recovery in Magsaysay reef will be very slow and may not occur without active intervention. In addition, the supply of larvae to surrounding reef areas in the Bolinao-Anda Reef Complex may be compromised, as computer model simulations suggest that Magsaysay reef is a source of coral larvae for the nearby reefs [69]. Therefore, the introduction of large numbers of competent *A*. *loripes* larvae to increase settlement and recruitment rates is an active management intervention designed to assist recovery of this coral population in Magsaysay reef. This intervention may also create a potential “spawning hot spot” or “spawning hub” in the future that may provide increased abundance of coral larvae with high genetic diversity for dispersal and restoration of adjacent degraded reef areas [66].

The initial settlement rates of *A*. *loripes* larvae on tiles in larval enhancement plots were relatively high, and comparable with those from the *A*. *tenuis* larval enhancement experiment, also conducted at the Magsaysay reef area a year earlier [17]. However, settlement patterns of *A*. *loripes* among tile surfaces was different from those recorded for *A*. *tenuis*. Initial settlement of *A*. *tenuis* larvae was significantly higher on tile sides with very few settlers on upper surfaces, whereas for *A*. *loripes*, larval settlement was not significantly different among tile surfaces. This suggests that larvae of these species may have different settlement orientation preferences, as recorded among other coral species [34, 70]. The initial settlement rates in the larval enhancement plots were about 70 times higher than the mean level of monitored natural acroporid recruitment in the study area over a two year period [17]. This pattern of enhanced larval settlement was similar to the ~100-fold increase in larval settlement recorded in the larval reseeding trial by Heyward et al. [24] at Ningaloo Reef in Western Australia, and much greater than the four-fold increase in settlement from the reseeding experiment of Edwards et al. [33] in Palau. These results suggest that larval density and benthic community composition can affect post-settlement survival and subsequent recruitment patterns. Therefore, to increase cost effectiveness, the optimal number of larvae introduced in a given reef area should be determined through density experiments to achieve maximum settlement and recruitment rates [71].

Survivorship of settled *A*. *loripes* spat on tiles showed a typical Type III survivorship curve. The proportion of surviving spat decreased significantly within the first month and continued to decline until 10 months when survivorship started to stabilise. That survivorship was similar to the survivorship pattern recorded for *A*. *tenuis* spat on tiles during the first 10 months after settlement [17], and in other laboratory cultured larvae settled on artificial substrata [72, 73]. These data show that the greatest bottleneck in coral post-settlement survivorship occurs when spat are small and most vulnerable during the first few weeks to months post-settlement [17, 19, 74, 75]. Possible causes for these declines in initial spat survivorship can be attributed to accidental or targetted grazing by fish and other macro-invertebrates, sediment smothering, and competitive interactions with other benthic organisms including microalgae and macroalgae, sponges and soft corals [75–79]. Post-settlement mortality is also intrinsically high among coral spat settled in controlled conditions in laboratory aquaria, even in the absence of predators, competitors, and other factors such as sediments [80]. Therefore, further investigations to determine the major drivers of post-settlement mortality are needed to increase the success of future direct larval enhancement interventions.

The survival rates of *A*. *loripes* corals that settled on the natural reef substrata and became visible at 10 months after settlement declined slowly over time but remained above 50% at the end of the 35 months monitoring period. This survivorship pattern contrasts with that recorded for *A*. *tenuis*, where there was no observed mortality of the faster growing *A*. *tenuis* recruits that settled on reef substrata from nine months to 35 months [17]. The different survivorship patterns of these two *Acropora* species may reflect intrinsic differences in their life-history characteristics, or may be related to size-escape thresholds [81, 82], with *A*. *tenuis* spat growing faster on average and attaining larger sizes more quickly than the *A*. *loripes* spat. Similar higher survivorship outcomes have been recorded when outplanting larger size sexually derived *Acropora* juvenile corals. In a study by Omori et al. [83], *A*. *tenuis* juveniles that had survived for 1.5 years in mid-water nurseries and reached 4 to 5 cm in diameter, had 89% survivorship after six months. In contrast, when smaller 1 cm diameter 6-month old *A*. *valida* juveniles were outplanted, a lower survivorship of 67% was recorded after six months [21].

After 35 months, the mean number of remaining *A*. *loripes* was 14 ± 2.9 per larval enhancement plot, which equates to an average of 1.8 coral colonies per m^2^ of available reef substrata. This density is slightly lower than the mean density of 2.3 *A*. *tenuis* colonies per m^2^ of available reef substrata recorded by dela Cruz and Harrison (2017), and partly reflects the slower growth and lower survivorship patterns for *A*. *loripes* colonies recorded in the present study. Edwards [9] suggested that for coral restoration interventions to be ecologically and economically cost-effective, there should be more than one coral surviving from every 10^4^ settled larvae. The total number of larvae that settled directly on the reef in this experiment could not be quantified because the microscopic larvae are too small to census and tend to have cryptic settlement on the complex reef surfaces. A total of 47 colonies survived after 35 months from an estimated 939,000 larvae added to the larval enhancement plots, which equates to one three-year-old colony per 20,000 larvae supplied. Based on the numbers of larvae settling on the tiles, it is likely that less than 50% of the larvae successfully settled during the five-day settlement period, hence the production rate probably exceeds the threshold suggested by Edwards [9].

This study has also provided the first data on growth rates of sexually produced *A*. *loripes* colonies. The growth rates of *A*. *loripes* that survived on the tiles and natural substrata (mean diameter 2.6 cm yr^-1^) was considerably slower than the growth rates of *A*. *tenuis* colonies grown from settled larvae at Magsaysay reef (mean diameter 5.1 cm yr^-1^) [17] and other sexually produced *Acropora* corals. Cultured *A*. *tenuis* larvae that settled on artificial substrata in an outdoor hatchery, and were subsequently transplanted to reef areas in subtropical Okinawa, Japan reached a mean diameter of 20 cm (4 cm yr^-1^) after 4 years of transplantation [84]. Similar growth rates were recorded for *A*. *millepora* corals cultured and maintained in nurseries for 3 years (4 cm yr^-1^ mean diameter), or transplanted onto reef areas (4.6 cm yr^-1^ mean diameter) [19, 85].

In contrast to sexually derived *A*. *tenuis* colonies that reached sexual maturity at 35 months when they had grown to a colony size larger than 12.5 cm mean diameter [17], none of the *A*. *loripes* colonies were gravid at 35 months age. This indicates that the minimum age at first reproduction in *A*. *loripes* might be at least four or five years, as predicted by Wallace [38]. Furthermore, the sizes of gravid colonies collected at the start of this study (7–15 cm mean diameter) show that some colonies can be sexually reproductive at sizes less than 12 cm mean diameter, which confirms previous information on the minimum size of reproduction of *A*. *loripes* [38, 86]. The delay in the onset of sexual reproduction of *A*. *loripes* colonies surviving at the end of this study suggests that size-dependent reproduction may not strictly apply in *A*. *loripes*, rather sexual reproduction could be influenced by both age and size [34, 86]. These differences in life-history patterns among *Acropora* species highlight the importance of choosing the best coral species for achieving specific coral restoration goals.

Some of the key challenges for coral restoration are to lower the production cost and to be adaptable to developing nations where most of the world’s coral communities occur and which are subjected to rapidly increasing anthropogenic threats [9, 87]. The low-cost mesh matting larval enclosures used in this study were made using widely available materials including organza (wedding veil) cloth, and were constructed using a standard non-electric sewing machine by local people. The nets were sturdy enough to withstand the underwater environmental conditions during the 5-day larval settlement period. The average production cost per *A*. *loripes* colony after 8 months ($17.41) and 35 months ($35.20) was slightly higher than the production costs for *A*. *tenuis* colonies at similar ages ($14.77 and $20.94, respectively) [17]. Chamberland et al. [29] recorded a production cost of $13 USD per 2.5 year old *Acropora palmata* colony outplanted onto reef areas two weeks after settlement onto clay tripods, and a higher cost of $325 USD per colony maintained in a land-based nursery. The production costs for the larval enhancement method are significantly lower in large part because settlers are not maintained in ex situ or in situ grow-out nursery installations over long periods of time. The overall cost-effectiveness of the larval enhancement technique will also increase as restored colonies become sexually reproductive and contribute larvae to the natural larval pool that can re-populate and colonize other available reef areas in future.

One of the advantages of the larval enhancement method using temporary mesh enclosures is the applicability and adaptability of the technique to various corals and reef types with different environmental conditions. In the present study, flat mesh matting was used to contain larvae on reef areas with relatively low topographical relief, and the reef experiences occasional strong wave action. For reef systems with more complex substrata, the enclosures can be modified to adapt to the contours of the reef. Furthermore, the availability of plankton nets with various mesh size openings makes the larval enhancement technique applicable to a wide range of spawning and brooding coral species that produce larvae of different sizes. Future larval enhancement interventions could be done without mesh enclosures by releasing competent larvae at optimal densities during periods of low tidal flows and calm weather and low wave action. This technique should also be tested in different reef regions around the world with different environmental conditions and topography, for comparison with other existing coral restoration techniques.

While high post-settlement mortality remains a key challenge for coral restoration using sexual production [34], the results of this study demonstrate that settlement and recruitment can be significantly enhanced via the provision of large numbers of sexually produced larvae. The larval enhancement technique has the advantage of settling larvae in situ on the reef and therefore surviving corals are likely to be well-adapted to local environmental conditions. This technique also avoids the need for large scale production of artificial settlement substrata and construction of in situ or ex situ coral nurseries for coral husbandry, and the extra effort and expense of manually transporting and attaching the coral recruits or colonies onto the reef substrata [5, 9]. In addition, direct larval provision onto degraded reefs has strong potential for scaling up restoration efforts to larger reef areas in future.
